# Evaluating The Expression of Self-Renewal Genes in Human Endothelial Progenitor Cells

**Published:** 2013-02-20

**Authors:** Hamid Bayat, Fardin Fathi, Habibollah Peyrovi, Seyed Javad Mowla

**Affiliations:** 1. Department of Molecular Genetics, Faculty of Biological Sciences, Tarbiat Modares University, Tehran, Iran; 2. Cellular and Molecular Research Center, Kurdistan University of Medical Sciences, Sanandaj, Iran; 3. Nanomedicine and Tissue Engineering Center, Shahid Beheshti University of Medical Sciences, Tehran, Iran

**Keywords:** Endothelial, Progenitor Cell, Marker, Stem Cells, Peripheral Blood

## Abstract

**Objective:**

Endothelial progenitor cells (EPCs) have a potential application for cell therapy, however, their biological nature is not well-understood. EPCs also possess some stemness features, such as their clonogenicity and differentiation capacity. The main aim of this study was to evaluate the expression of certain transcription factors regulating selfrenewal property of stem cells.

**Materials and Methods:**

In this experimental study, peripheral blood mononuclear cells were isolated from fresh human blood of several volunteers and were cultured in fibronectin- coated plates. EPCs were identified based on their morphology and growth characteristic. Then, the expression of some markers implicated in self-renewal capacity was assessed in the isolated cells using reverse transcription-polymerase chain reaction (RTPCR) and immunocytochemistry.

**Results:**

Expression of the cell surface markers, *CD31* and *CD34*, was determined by RT-PCR and immunocytochemistry. Furthermore, these cells had the ability for Di-ACLDL incorporation as well as attachment to lectin I. EPCs did not express the main stem cell markers, like *OCT4-A*, Nanog, and Sox2; nevertheless, they expressed the weaker pluripotent markers, including *OCT4B* and *OCT4-B1* spliced variants, such as *Nucleostemin* and *ZFX*. Furthermore, the expression of *Nucleostemin* and *ZFX* genes revealed a decreasing pattern from days 4^th^ to 11^th^.

**Conclusion:**

The main regulators of stem cell self-renewal genes, including *OCT4-A, Nanog,* and *Sox2* are not expressed in EPCs. Forced expression of these genes can elevate the stemness property and clinical application of EPCs.

## Introduction

Vasculogenesis was initially thought to be as the generation of new blood vessels by stem or progenitor cells, taking place only during embryonic development (1). The belief is later revised after Asahara et al. identified the endothelial progenitor cells (EPCs) in adult human peripheral blood (2). EPCs, a heterogeneous subpopulation of bone marrow mononuclear cells, are mobilized in response to ischemia and are able to differentiate into endothelial cells in ischemic organs (3). Conventionally, EPCs are recognized by the expression of both hematopoietic stem cell and endothelial cell markers (4). However their molecular criteria are not well-understood.

EPCs play an essential role in wound healing process as well as pathogenic conditions, such as tumor growth (5) and metastases (6). Dysfunction of EPCs occurs by various risk factors of coronary artery disease, such as aging, diabetes, hypercholesterolemia, and hypertension (7). Researchers have now begun to estimate the potential therapeutic use of EPCs in treatment of ischemic cardiovascular disease. Results indicate that EPCs are a safe and powerful tool in cell therapy of cardiovascular disease (8).

EPCs seem to have some features of stem cells, such as their clonogenicity and differentiation capacity (4). Similar to stem cells, they have the capacity of differentiation into several types of cells, including: cardiomyocytes, smooth muscle cells and endothelial cells (9). An important characteristic of stem cells is their self- renewal property, controlled by certain transcription factors which are exclusively expressed in stem cells (10). The main stem cell self-renewal regulatory genes are *OCT4, Nanog*, and *Sox2,* as well as *Nucleostemin* and *ZFX*. Evaluating the expression of these genes is one of the first steps in assigning the stemness property to stem cells.

While EPCs seem to be an important cell source for cell therapy, many aspects of their identity and characteristic still remain unclear. For example, a potential expression of selfrenewal genes in EPCs has not been comprehensively examined. In this study, we isolated endothelial progenitor cells from human peripheral blood and characterized the cellular and molecular identity of isolated cells as putative stem/progenitor cells.

## Materials and Methods

### Cell isolation and culture

In this experimental study, peripheral blood (100 ml) was obtained from some healthy volunteers. Mononuclear cells were separated from other components of the peripheral blood by density-gradient centrifugation on Histopaque 1077 (Sigma Aldrich, Belgium). Isolated cells were resuspended in EGM-2-MV Bullet Kit (Clonetics, USA) medium consisting of endothelial basal medium, 5% fetal bovine serum human epidermal growth factor (hEGF), vascular epidermal growth factor (VEGF), human growth factor-basic recombinant (hFGF-B), insulin-like growth factor 1 (IGF-1), ascorbic acid, and heparin. The peripheral blood mononuclear cells (PBMNCs) (1 × 107 per well) were then cultured in the coated (2.5µg per cm2 fibronectin; Sigma/Aldrich, Japan) six-well plates, and incubated in a 5% CO_2_ incubator at 37℃. After 4 days of culture, the medium containing non-adherent cells were collected, centrifuged, washed with phosphate-buffered saline (PBS), resuspended in fresh medium, and returned to the cell culture until 7^th^ day. The attached cells were cultured for 3 weeks. Culture medium was changed every 3 day. The experimental procedure was approved by the Ethical Board of Tarbiat Modares University.

### RNA extraction and RT-PCR

Total RNA was extracted on days 4^th^, 7^th^ and 11^th^ after seeding of mononuclear cells (MNCs) using the RNX Plus kit according to the manufacturer’s instruction (CinnaGen, Iran). The quality of RNA was estimated by gel electrophoresis as well as by optical density at 260 nm and 280 nm, respectively.

Therefore, 1 µg of the extracted RNA was treated with RNase-free DNase (Fermentas, Germany) and used for cDNA synthesis using 0.2 µg random hexamer and MMuLV Reverse Transcriptase (Fermentas, Germany) in a 20 µg reaction according to the manufacturer’s instructions. For each sample, a negative control, without addition of MMuLV, was also employed. PCR was performed using 2µg of synthesized cDNA with 1U of Taq polymerase (CinnaGen, Iran), 2.5 µl of 10x PCR buffer, 0.5 µl of 20 mM deoxy nucleotide tri-phosphates (dNTPs), 0.5 µl of each primer and deionized distilled water in a 25µl PCR reaction. The PCR amplification was carried out for 35 cycles for all genes, except for the internal control gene *GAPDH*, where the number of cycles was 30. The cycling conditions were as follows: 94℃ for 30 seconds, 55-64℃ (depending on primers used, Table 1) for 30 seconds, 72℃ for 45 seconds, and a final extension of 72℃ for 5 minutes. Each experiment was repeated at least three times.

**Table 1:** The sequences, annealing temperatures, and product sizes of the primers used to amplify genes of interest


Genes	Product size (bp)	Annealing temperature (˚C)	Forward primer sequence	Reverse primer sequence

Oct4-A	496	63.5	CTTCTCGCCCCCTCCAGGT	AAATAGAACCCCCAGGGTGAGC
Oct4-B	267	64	AGACTATTCCTTGGGGCCACAC	CTCAAAGCGGCAGATGGTCG
Oct4-B1	492	64	AGACTATTCCTTGGGGCCACAC	CTCAAAGCGGCAGATGGTCG
Sox2	426	60	ATGGGTTCGGTGGTCAAGTC	GTGGATGGGATTGGTGTTCTC
Nanog	470	59	ACCTATGCCTGTGATTTGTGG	AAGAGTAGAGGCTGGGGTAGG
Nucleostemin	747	60	CAGAGATCCTCTTGGTTGCAG	AATGAGGCACCTGTCCACTC
ZFX	465	60	TGATTCCAGGCAGTACCAAAC	TGACGAAAACCCTTACCACAC
CD31	578	59.5	AGGACATCCATGTTCCGAGA	TGAACCGTGTCTTCAGGTTG
CD34	219	56	TGAGCCTCTCACCTGTACTC	AGGAGCTGATCTGGGCTATG
KDR	180	61	CCCTGCCGTGTTGAAGAGTT	GGACAGGGGGAAGAACAAAA
VCAM1	375	58	AGAAATGCCCATCTATGTCC	CGGCATCTTTACAAAACCTG
Tie1	173	57	GCAAACTCTGCTGTCTAACC	GGATGCCCAGGATAGCTATG
GAPDH (1)	139	64	GCCCCAGCAAGAGCACAAGA	TAGGCCCCTCCCCTCTTCAA
GAPDH (2)	224	60	AGGGTCTCTCTCTTCCTCTTGTGCTC	CCAGGTGGTCTCCTCTGACTTCAACA


### Immunocytochemistry

Immunocytochemistry was performed on EPCs at 7^th^ day of seeding. Briefly, the cells were fixed with 4% paraformaldehyde for 20 minutes at room temperature. Cells were then washed with PBS containing 1% bovine serum albumin (BSA), 0.02% Triton X-100 and 10% rabbit serum while incubated with blocking solution for 30 minutes at room temperature. The cells were then incubated with primary polyclonal antibodies (anti-*CD31*, 1:40; anti-*CD34*, 1:50 from Dako, USA and anti-*OCT4*, 1:50 from Santa Cruz Biotechnology, USA) for 2 hours at 37℃. After washing with PBS, the cells were incubated with the secondary antibodies (dilution 1:100) as follows: anti-mouse IgG conjugated with cy3 (Cmicon, CA, USA), anti-goat IgG conjugated with FITC (Jackson Immuno Research Labs, USA), and anti-rabbit IgG conjugated with fluorescein isothiocyanate (FITC) (Sigma-Aldrich, USA), respectively, for attachment to anti-*CD31*, anti-*CD34* and anti-*OCT4* for 45 minutes at 37℃ followed by washing further three times with PBS at room temperature.

Finally, the immunoreactivity was analyzed with a fluorescent microscope (Nikon TE300, Japan).In a similar approach, the negative control were prepared, except for the omission of primary antibodies.

## Results

### Isolation of endothelial progenitor cells

**Fig 1:**
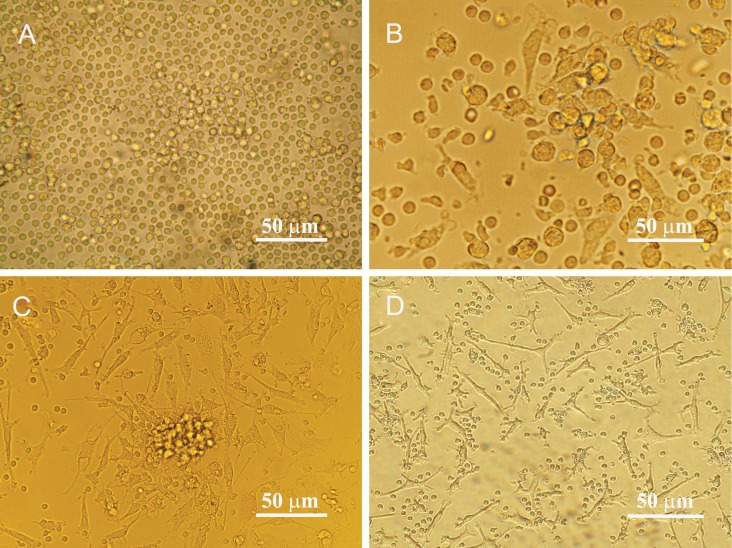
Morphological features of isolated endothelial progenitor cells before and after differentiation. A. Mononuclear cells isolated from human peripheral blood appeared small and round. B. Most cells were attached to the plates coated with human fibronectin (day 4). The attached cells gradually exhibited a spindle-shaped, endothelial cell-like morphology on day 7^th^ C. and day14^th^ D. after seeding.

On the 7^th^ day of MNCs culture, ex vivo expanded EPCs showed two kinds of morphology, including spindle-shape cells and round cells, yet some EPC colonies were observable. With continued culture, the number of round cells decreased and spindleshape cells become dominant cell type (Fig 1).

**Fig 2:**
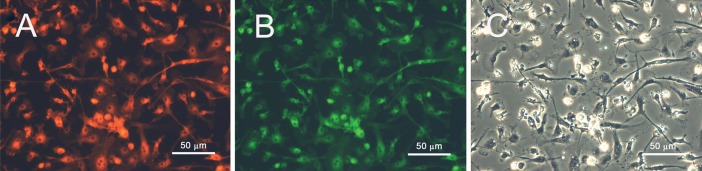
Fluorescence images of isolated endothelial progenitor cells on day 7^th^ after seeding showing positive staining for: A. acetylated LDL (DiI-acLDL) and B. lectin. C. The phase contrast counterpart of A and B.

**Fig 3:**
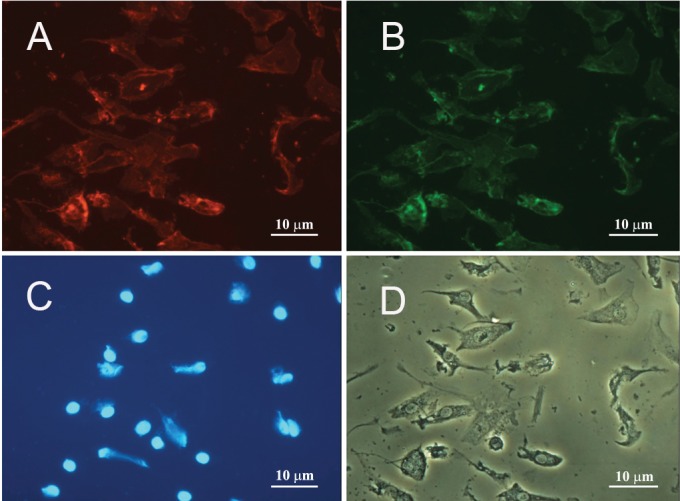
A. Immunocytochemistry of EPCs double-stained with anti-CD31, and B. anti-CD34 antibodies. C. Cells' nuclei were visualized with DAPI . D. The same phase contrast view of the cells in A-C.

**Fig 4:**
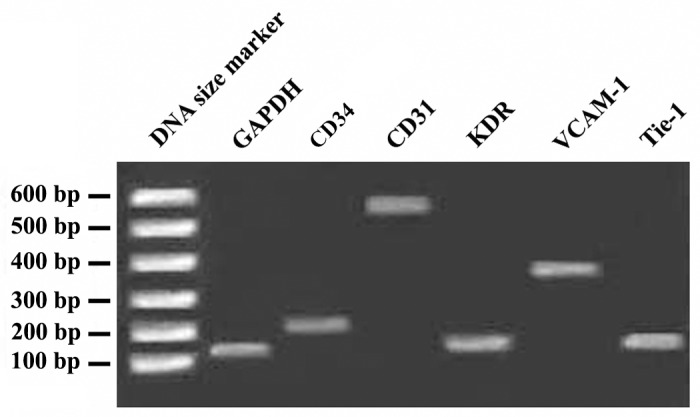
ART-PCR analysis of the expression of endothelial markers in endothelial progenitor cells (EPCs) at day 7^th^ after seeding. As it is evident, EPCs express CD34, *CD31*, KDR, VECAM-1 and Tie-1 genes. GAPDH was used as an internal control.

By the 7^th^ day, the identity of EPCs was confirmed through double staining for Di-AC-LDL and lectin binding (Fig 2). The obtained data was further validated by immunocytochemistry results revealing that the isolated cells express *CD31* and *CD34*, cell surface markers (Fig 3). Moreover, further characterization of EPCs was carried out by studying the expression of *CD34, KDR, VCAM-1, CD31* and *Tie-1* by RTPCR, where all of these markers were expressed in isolated cells (Fig 4). All together, our data confirmed the genuine nature of the isolated cells as EPCs.

### Evaluating the expression of stem cell marker genes in EPCs

**Fig 5:**
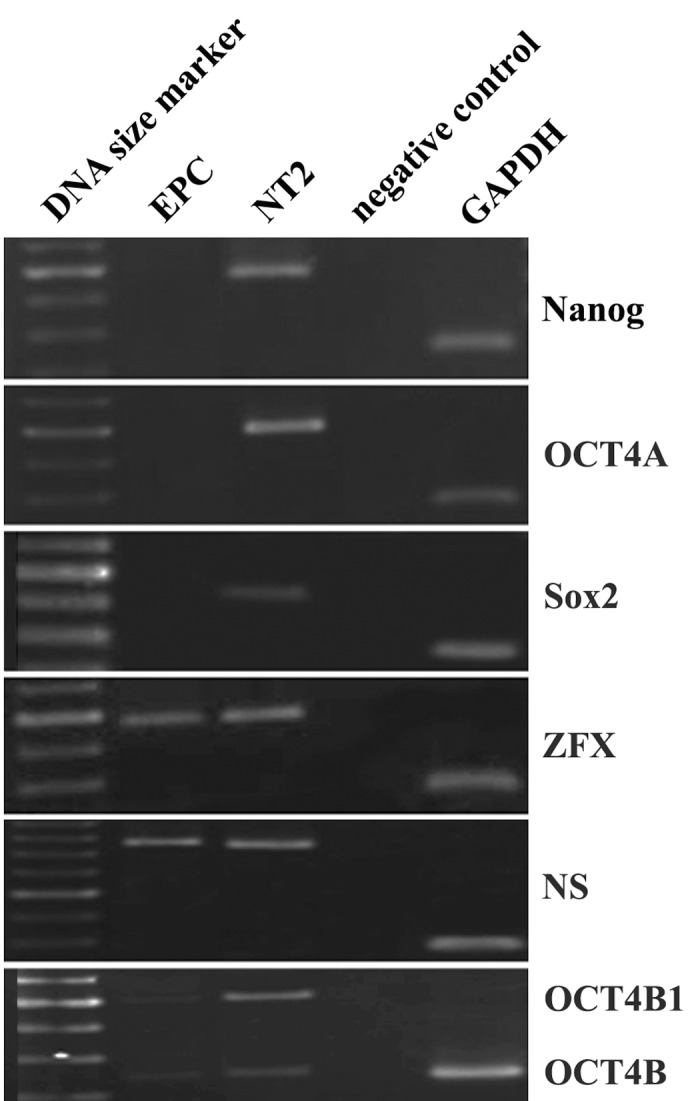
RT-PCR analysis of the expression of some selfrenewal genes in EPCs on day 7^th^ after seeding. Note that EPCs do not express OCT4A variant, Nanog, and Sox2, but express *Nucleostemin (NS)*, *ZFX*, OCT4B, and OCT4B1 variants. The embryonic carcinoma cell line *NT2* was used as a positive control for pluripotency. *GAPDH* was used as an internal control.

RT-PCR analysis of EPCs on 7^th^ day of culture revealed that the main regulators of self-renewal property in embryonic stem cells, namely *OCT4A*; *Nanog*; and *Sox2* are not expressed in EPCs (Fig 5). However, the adult stem cell markers, *Nucleostemin* and *ZFX*, and to a lesser extent the other spliced variants of *OCT4*, like *OCT4B* and *OCT4B1* were expressed in the cells (Fig 5). The expression of genes was compared to that of *NT2*, a human carcinoma cell line used as a positive control. Moreover, the expression of *GAPDH*, a housekeeping gene, was monitored in all experiments as an internal control. Also, a no-reverse transcription (no- RT) control was accompanying all PCR experiments, as a negative control.

**Fig 6:**
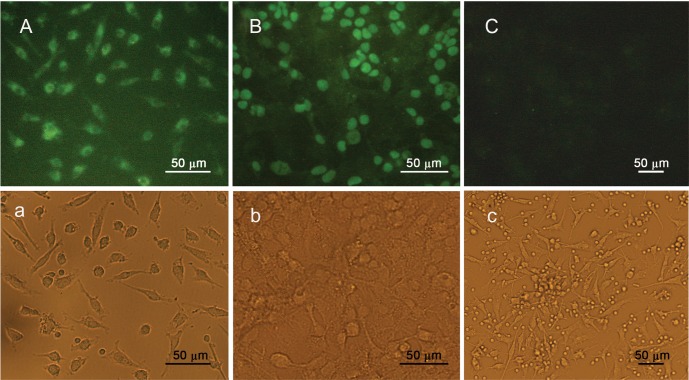
Sub-cellular localization of OCT4 in EPCs was stained with a polyclonal antibody against C-terminal domain of OCT4. A. Immunocytochemistry revealed a cytoplasmic immunoreactivity for EPCs. B. NT2 cells were used as a positive control and demonstrated a primarily nuclear immunoreactivity. C. Negative control slide, all the conditions were the same as A and B, except for the elimination of the primary antibodies. a, b, and c depict the phase-contrast counterparts of A, B and C, respectively.

Immunocytochemistry analysis of the subcellular distribution of *OCT4* demonstrated a cytoplasmic signal in EPCs, with no immunoreactivity presence within the nuclei of the cells. The observations further confirmed the expression of *OCT4B* and/or *OCT4B1* variants which are localized within the cytoplasm of the cells. On contrary, the *OCT4* immunoreactivity was primarily visualized within the nuclei of the pluripotent cell line *NT2*, which is consistent with the expression of *OCT4A* variant within these cells. As expected, no staining signal was detected in the negative control, in which all of the conditions were kept the same, except for the omission of the primary antibody (Fig 6).

**Fig 7:**
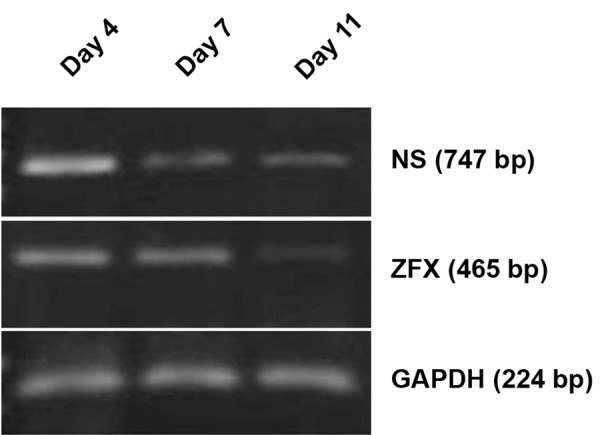
Changes in NS and ZFX genes expression during differentiation of EPCs. As it is evident, there is a decline in the expression of NS and ZFX in the course of cell differentiation from the day 4^th^ to day 11^th^ of cell seeding.

The expression levels of the Nucleostemin and *ZFX* genes seem to be down-regulated from days 4^th^ to 11^th^. For Nucleostemin and *ZFX* genes, an apparent decrease in the expression level was observed on days 4^th^ and 7^th^ of the seeding (Fig 7).

## Discussion

EPCs in the human peripheral blood are a potential source of stem cells to promote angiogenesis in ischemic tissues (3). However, the cells have not yet been well characterized and many questions remain to be answered about their nature and molecular characteristics.

In the present study, the isolation of EPCs was carried out by an ex vivo expansion of a heterogeneous PBMC fraction. In this method of cell isolation, the number of isolated cells is higher than other methods (11). In this regard, using magnetic beads for EPCs evaluation may influence the viability of the cells. It can also preferentially exclude some cells with endothelial potential, thereby decrees the number of cells within an isolation population (12). Moreover, it seems that cultivated EPCs in this method are more heterogeneous. After 7 days of culture, the attached cells have the ability of Dil-AC-LDL incorporation, lectin banding, as well as the expression of *CD34*, *CD31*, *VCAM-1, Tie-1* and *KDR* genes. All of the mentioned properties indicate an endothelial identity for the expanded cells.

After isolation and expansion of EPCs, the expression of self-renewal genes, like *OCT4, Nanog*, and *Sox2* were evaluated by RT-PCR .These genes form the core of main regulatory network that induce the expression of genes maintaining the pluripotency of embryonic stem cells (ESCs), and also suppress some genes inducing differentiation (10). Our results revealed that none of these genes is expressed in EPCs; therefore, we suggest that EPCs do not have self-renewal properties similar to ES cells. This finding is in accordance with some recent reports that suggest *OCT4A* is exclusively expressed in pluripotent ES/EC cells (13). The absence of *OCT4A* expression in already reported in some adult stem cells, such as peripheral blood mononuclear cells (14) and rat bone marrow stromal stem cells (15).

However, our data is in contrast with Romagnani et al. who have previously reported the expression of *OCT4* and Nanog in a subpopulation of EPCs, *CD14* + *CD34* LOW (16). Interestingly, all the previous publications on *OCT4A* expression in different cell types is doubted with the presence of several expressed pseudogenes of *OCT4*, as well as with the presence of two other splicing variants of the gene (*OCT4B* and *OCT4B1*). The very high sequence conservation between *OCT4A*, its pseudogenes, and splicing variants could be likely a source of misinterpretation of RT-PCR and immunostaining experiments, as genuine *OCT4A* expression. Therefore, here we designed specific primers to avoid pseudogenes amplification, and also to discriminate *OCT4* alternatively spliced variants (13). Our data revealed no expression of *OCT4A*, but expression of *OCT4B* and *OCT4B1* spliced variants in endothelial progenitor cells. While *OCT4A* has a well-known critical role in maintaining the pluripotency of embryonic stem cells, little is known about the functional role of *OCT4B* and *OCT4B1* variants in pluripotent and non-pluripotent cells. However, it has been reported recently that *OCT4B* and *OCT4B1* are also expressed in non-pluripotent cells, where their proteins are localized within the cytoplasm of the cells (13, 17, 18).

In addition, we also detected for the first time the expression of *ZFX*, a newly identified self-renewal regulatory gene, in EPCs. *ZFX* has been shown to be expressed in both embryonic and adult (hematopoietic) stem cells, likely an anti-apoptotic role in these cells (19, 20). Hematopoietic stem cells are one of the major sources of EPCs in peripheral blood. Therefore, it is concluded that the expression of *ZFX* in this pathway continues until completion of the differentiation of EPCs which it likely seems to have the same role in self-renewal of EPCs. Furthermore, our results demonstrated that EPCs express *Nucleostemin (NS)* gene and that its expression was down-regulated from days 4^th^ to 11^th^. NS is highly expressed in bone marrow stromal cells, and the expression is suddenly turned off with the induction of neural differentiation in the cells (15). Based on recent reports, NS was hypothesized to be a proliferating factor in stem cells, where it regulates cell cycle proceeding (21).

## Conclusion

Our data revealed that EPCs express endothelial markers, an indication that they are in a late stage of cell differentiation. The data also revealed that none of the main ES-specific regulatory genes *OCT4A*, *Sox2* and *Nanog* is expressed in EPCs; a finding that indicates these cells have a limited self-renewal capacity compared to embryonic stem cells. The latter finding suggests that the cells could be considered as a safer source compared to ES cells for cell therapy, in terms of the likelihood of teratoma generation.
